# Chronic Methamphetamine Administration Causes Differential Regulation of Transcription Factors in the Rat Midbrain

**DOI:** 10.1371/journal.pone.0019179

**Published:** 2011-04-25

**Authors:** Irina N. Krasnova, Bruce Ladenheim, Amber B. Hodges, Nora D. Volkow, Jean Lud Cadet

**Affiliations:** 1 Molecular Neuropsychiatry Research Branch, DHHS/NIH/NIDA Intramural Research Program, Bethesda, Maryland, United States of America; 2 Department of Psychology, Morgan State University, Baltimore, Maryland, United States of America; 3 National Institute on Drug Abuse (NIDA), National Institutes of Health (NIH), U.S. Department of Health and Human Services (DHHS), Bethesda, Maryland, United States of America; Okayama University Graduate School of Medicine, Dentistry and Pharmaceutical Sciences, Japan

## Abstract

Methamphetamine (METH) is an addictive and neurotoxic psychostimulant widely abused in the USA and throughout the world. When administered in large doses, METH can cause depletion of striatal dopamine terminals, with preservation of midbrain dopaminergic neurons. Because alterations in the expression of transcription factors that regulate the development of dopaminergic neurons might be involved in protecting these neurons after toxic insults, we tested the possibility that their expression might be affected by toxic doses of METH in the adult brain. Male Sprague-Dawley rats pretreated with saline or increasing doses of METH were challenged with toxic doses of the drug and euthanized two weeks later. Animals that received toxic METH challenges showed decreases in dopamine levels and reductions in tyrosine hydroxylase protein concentration in the striatum. METH pretreatment protected against loss of striatal dopamine and tyrosine hydroxylase. In contrast, METH challenges caused decreases in dopamine transporters in both saline- and METH-pretreated animals. Interestingly, METH challenges elicited increases in dopamine transporter mRNA levels in the midbrain in the presence but not in the absence of METH pretreatment. Moreover, toxic METH doses caused decreases in the expression of the dopamine developmental factors, *Shh*, *Lmx1b*, and *Nurr1*, but not in the levels of *Otx2* and *Pitx3*, in saline-pretreated rats. METH pretreatment followed by METH challenges also decreased *Nurr1* but increased *Otx2* and *Pitx3* expression in the midbrain. These findings suggest that, in adult animals, toxic doses of METH can differentially influence the expression of transcription factors involved in the developmental regulation of dopamine neurons. The combined increases in *Otx2* and *Pitx3* expression after METH preconditioning might represent, in part, some of the mechanisms that served to protect against METH-induced striatal dopamine depletion observed after METH preconditioning.

## Introduction

Methamphetamine (METH) is a psychostimulant that is abused throughout the world. Acute administration of the drug causes behavioral changes that are secondary to activation of dopaminergic systems located in various brain regions [1]. Chronic abuse of METH causes adverse neuropsychiatric effects which include addiction, psychosis and cognitive impairments (reviewed in [2]) and, possibly, Parkinsonism [3]. Some of the cognitive abnormalities are thought to be related to METH-induced neurodegenerative changes in the brains of human addicts [4]. Of significant concern are the findings from imaging and postmortem studies describing decreases in the density of striatal dopamine transporters (DAT), reductions in tyrosine hydroxylase (TH) levels as well as decreases in the concentrations of dopamine (DA) in the brains of chronic METH abusers [5], [6]. In fact, these abnormalities might reflect damage to DA neurons and the possibility that dysfunctional DA neurons could lead to the appearance of neurological syndromes over time [7], [8].

In preclinical studies, injections of moderate-to-large doses of METH cause depletion of DA as well as loss of DAT and TH in the striatum of rodents and non-human primates [8]. These changes occur without any clear evidence of DA neuronal death in the midbrain (reviewed in [7], [8]). On the other hand, injections of increasing but nontoxic METH doses provide protection against subsequent challenges with larger toxic doses of the drug [9]–[12]. We recently termed this process, METH preconditioning [9], because of its similarities to other neuroprotective preconditioning paradigms [13]. Although it has become clear that METH-induced depletion of DA and decreases in DAT and TH expression in the striatum are dependent on toxic processes such as the production of free radicals, generation of DA quinones, glutamate-mediated formation of nitric oxide, and temperature dysregulation (reviewed in [8]), much remains to be done to clarify the mechanisms responsible for the lack of cell death of midbrain DA neurons and to explain the progressive recovery of DA levels in METH-treated rodents [14], [15]. We thought that repeated injections of METH might generate a tolerant state that imbues midbrain DA cells with a certain degree of resistance against the axonal retrograde degeneration that is observed after intrastriatal injections of 6-hydroxydopamine (6-OHDA) [16], [17] since METH and 6-OHDA share basic mechanisms of toxicity [18]. We also thought that the development of this tolerant state, that guards against midbrain neuronal death, might be secondary to METH-induced recapitulation of molecular events that are engaged in the generation, promotion, and protection of DA neurons during developmental stages in utero [19], [20]. Specifically, the development of mesostriatal dopaminergic pathways is coordinated by the interactions of diverse differentiating and maintenance signals that are being dissected by various research groups [20], [21]. These include transcription factors OTX2, WNT1, SHH, FGF8, LMX and MSX that are involved in regulating the early development of DA neurons [22]–[26]. Other transcription factors of interest are NURR1 and PITX3 which participate in the induction and maintenance of DA neurons and cooperate to promote their maturation [27]–[30]. Because some of these transcription factors continue to be expressed in the adult CNS, we reasoned that they might also mediate the preservation of the dopaminergic phenotype and survival of DA neurons in the adult brain. Herein, we report, for the first time, that challenges with toxic doses of METH caused significant changes in the expression of transcription factors that are involved in the development of midbrain dopaminergic neurons. This differential regulation might be important to the maintenance of midbrain DA neurons in the presence of METH-induced degeneration of striatal DA terminals.

## Materials and Methods

### Animals

Male Sprague-Dawley rats (Charles River Laboratories, Raleigh, NC) weighing approximately 350–400 g were habituated for one week prior to drug treatment. Animals were housed in polyethylene cages containing hardwood bedding in a temperature-controlled room with a 12 hour light∶dark cycle and free access to food and water. All animal procedures were performed according to the National Institutes of Health *Guide for the Care and Use of Laboratory Animals* and were approved by the Animal Care and Use Committee of the National Institute on Drug Abuse, Intramural Research Program. The research was conducted under Animal Study Protocol #09-CNRB-25.

### Drug Treatment and Tissue Collection

After habituation, rats were injected with saline or with progressively higher doses of *d,l*-METH hydrochloride for two weeks as described in Table S1. The saline-pretreated group was further divided into three subgroups. Rats from the first subgroup were given saline and challenged twice with saline during the third week of the experiment (SSS). Rats form the second subgroup were challenged with METH (5 mg/kg×6, given 1 hour apart) (SSM). The third subgroup received two challenges of the same doses of METH within three days (SMM). Animals pretreated with METH were also challenged with METH (MMM). Clinical studies have indicated that most human METH addicts initially use low doses of the drug, taken at variable intervals; this is followed by progressive dose increases and subsequent escalation to repeated binges, with consumption of about 20 g of METH per week separated by variable lengths of abstinence [31]–[33]. Therefore, to better approximate METH abuse patterns reported in humans, we administered METH to rats according to a regimen of escalating METH doses followed by multiple drug binges. Models similar to this one have been previously used by several groups of investigators who study METH toxicity [9]–[11], [34]–[37].

Rats were weighed three times per week during the pretreatment period and both challenge days to ensure proper dosing. Tympanic temperatures were taken 30 minutes prior to the first injection and 30 minutes after every other injection on the second challenge day. The animals were euthanized 14 days following the METH challenge by decapitation. Their brains were then quickly removed, dorsal striata and midbrain regions were dissected over ice, snap frozen on dry ice, and stored at −80°C until used in biochemical experiments.

### HPLC

For monoamine analysis, the dorsal striata of 5–8 mice per group were homogenized in 0.01 M HClO_4_ and centrifuged at 14000× g for 15 min. DA, 3,4-dihydroxyphenylacetic acid (DOPAC) and homovanillic acid (HVA) levels were analyzed in the striatal extracts using HPLC with electrochemical detector as described previously [38] and expressed as pg/mg of tissue weight.

### qRT-PCR

Total RNA extracted from a midbrain region that encompasses the ventral tegmental area and substantia nigra of the rat was used to analyze the expression of genes of interest by qRT-PCR as previously described [34], [39], [40]. In brief, unpooled total RNA obtained from 5–7 rats per group was reverse-transcribed with oligo dT primers and Advantage RT for PCR kit (Clontech, Palo Alto, CA). PCR experiments were performed using LightCycler FastStart DNA Master SYBR Green I kit (Roche, Indianapolis, IN) according to manufacturer's protocol. Sequences for gene-specific primers corresponding to PCR targets were obtained using LightCycler Probe Design software (Roche). The primers were synthesized and HPLC-purified at the Synthesis and Sequencing Facility of Johns Hopkins University (Baltimore, MD). PCR values were normalized using 18S rRNA and quantified. The results are reported as fold changes calculated as the ratios of normalized gene expression data of each group in comparison to the SSS group.

### Western Blot analysis

Western blot analyses were performed as previously published [41], [42]. In brief, dorsal striatal and midbrain samples were washed with ice-cold 0.1 M PBS, homogenized in lysis buffer (0.01 M Tris-HCl, pH 7.6, 0.1 M NaCl, 0.001 M EDTA, 100 µg/ml PMSF and 1 µg/ml aprotinin) and then centrifuged at 15000× g for 30 min. Protein concentrations were determined with BioRad D_c_ Protein assay (BioRad, Temecula, CA). 20 µg of total protein were electrophoresed on 10% SDS-polyacrylamide gels and then transferred to Hybond-P™ membrane (GE Healthcare, Piscataway, NJ). The membranes were blocked and then immunolabeled with antibodies against DAT (1∶1000), TH (1∶10000), Pitx-3 (1∶1000) (all from Millipore, Billerica, MA) and Nurr1 (1∶1000; ThermoFisher Scientific, Waltham, MA) at 4°C overnight. Immune complexes were detected with HRP-labeled second antibody and ECL+ chemiluminescence reagents (GE Healthcare). To confirm equal protein loading, blots were stripped and reprobed with anti-α-tubulin antibody (1∶3000; Sigma, St. Louis, MO) for 2 hours at room temperature. Signal intensity was measured using densitometric analysis (Image Station 4000 MM Pro; Carestream Health, Inc., Rochester, NY) and quantified using Carestream Molecular Imaging Software (version 5.0.2.30, Carestream Health, Inc.).

### Statistical Analyses

All data are presented as means ± SEM. Statistical analysis of the experimental data on body temperature and weights was performed using two-way ANOVA for repeated measures followed by a pairwise multiple comparison procedure (Tukey's test) to identify differences between the groups or sessions, respectively (SigmaStat software, http://www.systat.com). Statistical analysis of HPLC, qRT-PCR and Western blot data was performed using one-way ANOVA for repeated measures followed by Fisher's protected least significant difference (PLSD) (StatView 4.02, SAS Institute, Cary, NC). The null hypothesis was rejected at *p*<0.05.

## Results

### METH-induced changes in body weights and temperature

**Figure 1:**
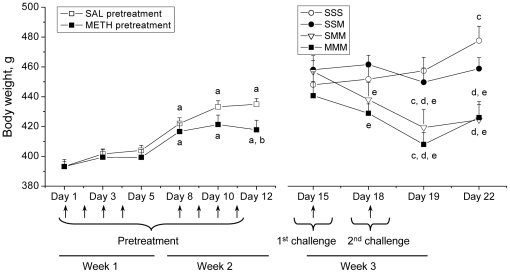
Methamphetamine administration causes decreases in body weights in the rat. (A) Rats pretreated with saline and METH showed increases in their body weights on Days 8, 10 and 12 in comparison to Day 1, with the METH group gaining significantly lower weights by Day 12. (B) On day 15, The saline-pretreated group was then divided into 3 groups that received saline challenges (SSS) or a single (SSM) or two METH challenges (SMM). The SSS group continued to gain weight after two saline challenges given on Day 22. However, rats challenged once with METH (SSM group) did not show significant changes in their body weights. In contrast, animals challenged twice with METH (SMM and MMM groups) showed reduction of their body weights in comparison to SSS and SSM groups on Days 19 and 22. The arrows indicate the day of saline or METH injections. Values are expressed as means ± SEM. (N = 6–32 animals per group). Key to statistics: ^a^ p<0.05 in comparison to Day 1; ^b^ p<0.05 in comparison to saline-pretreated group; ^c^ p<0.05 in comparison to Day 15; ^d^ p<0.05 in comparison to SSS group; ^e^ p<0.05 in comparison to SSM group.

The effects of METH pretreatment and challenges on body weights in rats are shown in Figure 1. There were no significant differences in body weights between saline- and METH-pretreated groups before initiating the pretreatments (Fig. 1). Rats in both groups showed significant increases in their body weights at the end of the pretreatment period. However, METH-pretreated rats gained significantly less weight than saline-pretreated group, +6.3% vs +10.6%, respectively (Fig. 1). There were no significant differences in body weights between the four groups of rats prior to challenges on Day 15 (Fig. 1). The SSS group showed increases in their body weights at the end of challenges. The single METH challenge (SSM group) did not induce any significant changes in body weights during the post-injection week. In contrast, the two METH challenges caused decreases in rat body weights independent of the type of pretreatment (−8.4% for SMM group and −7.5% for MMM group) (Fig. 1). Our findings of the weight loss in rats challenged with METH are consistent with the observations of Davidson [43] who reported decreased body weights in rats treated with METH via mini-pumps. These data are also in accord with our previous observations of weight loss in rats that self-administered METH [41]. In addition, our findings are in agreement with significant anorexia and weight loss reported in human METH abusers [33], [44], [45].

**Figure 2:**
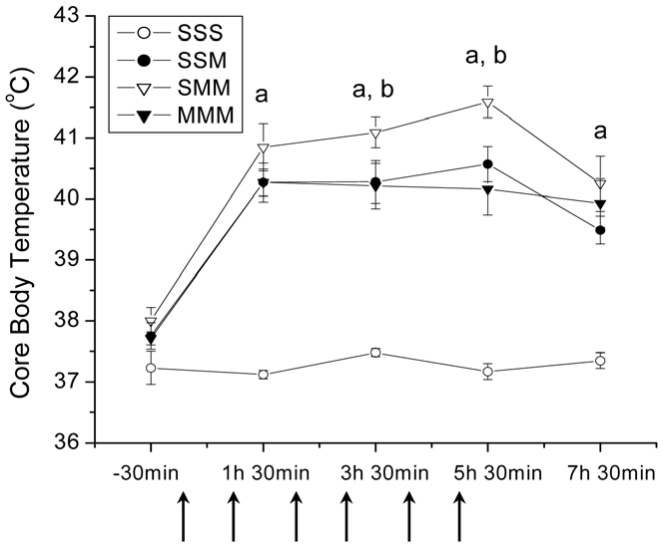
METH treatment induced increases in tympanic temperatures in rats. Temperatures were measured in all treatment groups after injections of either saline or METH or saline challenges (shown by arrows). Rats from the first group were given saline and challenged twice with saline during the third week of the experiment (SSS). Rats from the second group were challenged with METH (5 mg/kg×6, given 1 hour apart) (SSM). The third group received two challenges of the same doses of METH within three days (SMM). Animals from the fourth group pretreated with METH were also challenged with the drug (MMM). Values are expressed as means ± SEM. N = 6–10 animals per group. Key to statistics: ^a^ p<0.001 versus SSS group; ^b^ p<0.05 versus SSM and MMM groups.

Figure 2 shows the changes in rat body temperatures caused by the second METH challenges. There were no significant differences in body temperatures between the groups before METH injections. After the first injection, all METH-treated animals experienced significantly higher body temperatures than rats treated with saline. These increases in body temperature persisted throughout the time of observation. Interestingly, rats pretreated with saline and challenged with METH showed significantly higher core body temperature in response to the second drug challenge (SMM group) in comparison to SSM and MMM groups (Fig. 2).

### METH pretreatment protects against METH-induced depletion of striatal monoamines

Table 1 shows the effects of challenges with toxic doses of METH on DA, DOPAC, and HVA levels in the striatum at 14 days after treatment. In agreement with previous studies [8], repeated injections of toxic doses of METH during a single day caused substantial depletion of DA and its metabolites in saline-pretreated animals. An identical challenge given three days later did not cause any further reduction in DA nor its metabolites. As previously reported by us and others [9], [10], [12], METH pretreatment provided protection against DA and DOPAC depletion in the striatum.

**Table 1:** Effects of METH preconditioning and challenges on the levels of DA, DOPAC and HVA in the rat striatum.

Group	DA (pg/mg of tissue)	DOPAC (pg/mg of tissue)	HVA (pg/mg of tissue)
SSS	6716.1±319.4	826.1±18.2	428.4±42.3
SSM	3360.8±460.1^a^	534.5±46.3^a^	353.6±39.1
SMM	3360.8±460.1^a^	513.1±63.2^a^	338.1±24.7
MMM	5065.5±795.4^b^	752.6±97.9^b^	420.7±84.5

Values represent means ± SEM (N = 5–8 per group).

a *p*<0.01 in comparison to SSS group.

b *p*<0.05 in comparison to SSM and SMM groups.

### Effects of METH on striatal TH and DAT protein levels

**Figure 3:**
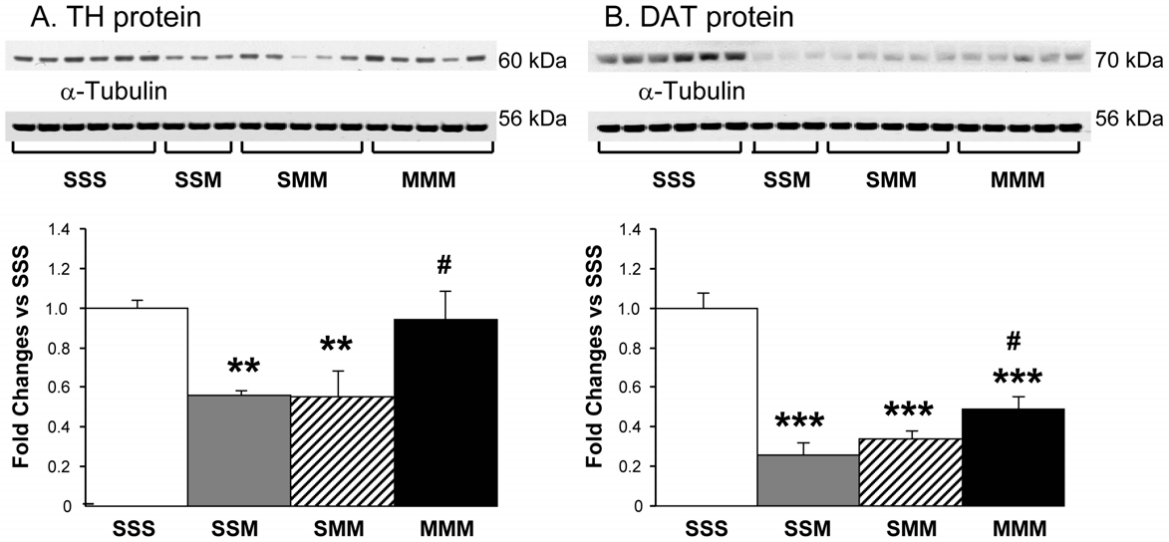
METH challenges caused decreases in TH and DAT protein levels in the striatum. METH preconditioning resulted in complete protection against decreases in TH protein levels (A). In contrast, METH preconditioning was only partially protective against METH toxic effects on DAT protein expression (B). The groups are as described in the legend to Fig. 2. The quantification data represent fold changes (means ± SEM) in comparison to the saline-pretreated group challenged with saline (SSS group). N = 3–6 animals per group. Keys to statistics: **, *** p<0.01, 0.001, respectively, in comparison to the SSS group; # p<0.05; in comparison to the SSM group (panels A and B).

The effects of METH on TH and DAT protein levels in the rat striatum are presented in Fig. 3. A single-day toxic METH challenge caused significant decreases in TH protein expression in rats euthanized two weeks after drug injections (Fig. 3A). An additional METH challenge, given three days later, did not potentiate the toxicity of the drug. Similar to its effects on DA levels, METH preconditioning caused protection against decreases in TH protein expression in the striatum.

METH-induced changes on striatal DAT expression are shown in Fig. 3B. The single and double METH challenges caused similar decreases in striatal DAT expression in the animals pretreated with saline. METH challenges also decreased DAT protein levels in METH-pretreated rats. However, the values in the latter group were higher than those measured in the saline-pretreated METH-challenged group (SSM).

### Effects of METH on midbrain TH and DAT mRNA and protein levels

**Figure 4:**
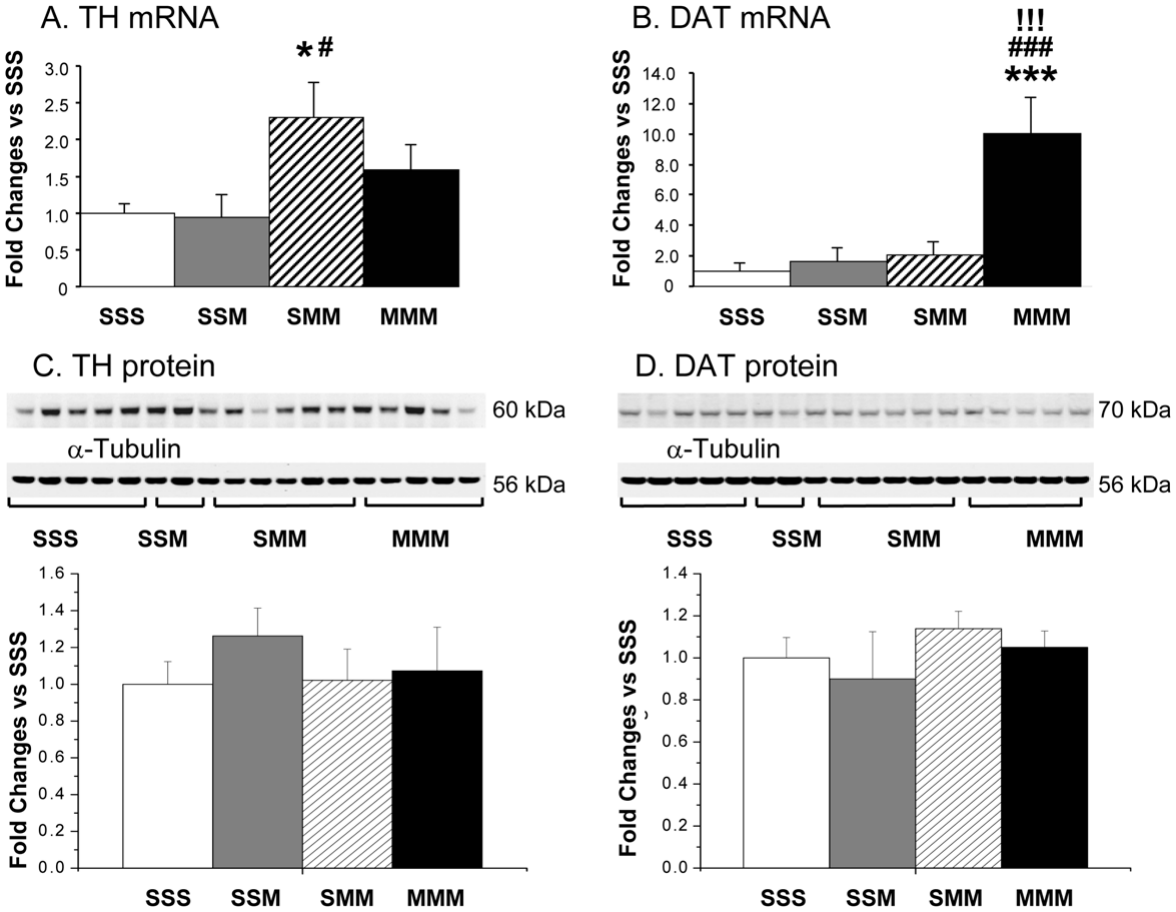
Effect of METH treatment on TH and DAT mRNA and protein levels in the midbrain. METH injections caused increases in TH mRNA only in the saline-pretreated group challenged twice with toxic doses of the drug (A). METH toxic challenges induced increases in DAT mRNA only in METH-pretreated rats (B). Total RNA was obtained from 5–6 animals per group, the mRNA expression was measured individually and normalized to 18S rRNA levels. The METH challenges caused no significant changes in the expression of TH (C) and DAT (D) protein levels. The values represent means ± SEM in comparison to the saline-pretreated group challenged with saline (SSS group). N = 3–5 animals per group. Keys to statistics: *, **, *** p<0.05, 0.01, 0.001, respectively, in comparison to the SSS group; #, ##, ### p<0.05, 0.01, 0.001, respectively, in comparison to the SSM group; !, !!, !!! p<0.05, 0.01, 0.001 respectively, in comparison to the SMM group.

Figure 4 shows *Th* and *Dat* mRNA levels in the midbrain of the animals pretreated with saline or METH before the drug challenges. Similar to the results of a previous report on the effects of amphetamine on *Th* mRNA [46], a single METH challenge associated with decreased striatal TH protein caused no changes in midbrain *Th* mRNA levels (Fig. 4A). However, two METH challenges caused increases in *Th* mRNA levels in the midbrain of rats pretreated with saline. METH pretreatment followed by METH challenges was also associated with normal levels of *Th* mRNA expression (Fig. 4A). Neither a single nor two METH challenges resulted in significant changes in *Dat* mRNA levels in rats pretreated with saline. Unexpectedly, there were marked METH-induced increases in *Dat* mRNA expression in the METH preconditioned animals (Fig. 4B). The effects of METH on TH and DAT protein levels in the midbrain are shown in Fig. 4C, D. The drug did not cause any significant changes in their expression in any of the METH-treated groups.

### Effects of METH on midbrain mRNA levels of differentiating factors of DA neurons

#### Effects of METH on midbrain *Otx2*, *Wnt*, *Shh*, and *Fgf8* mRNA levels

**Figure 5:**
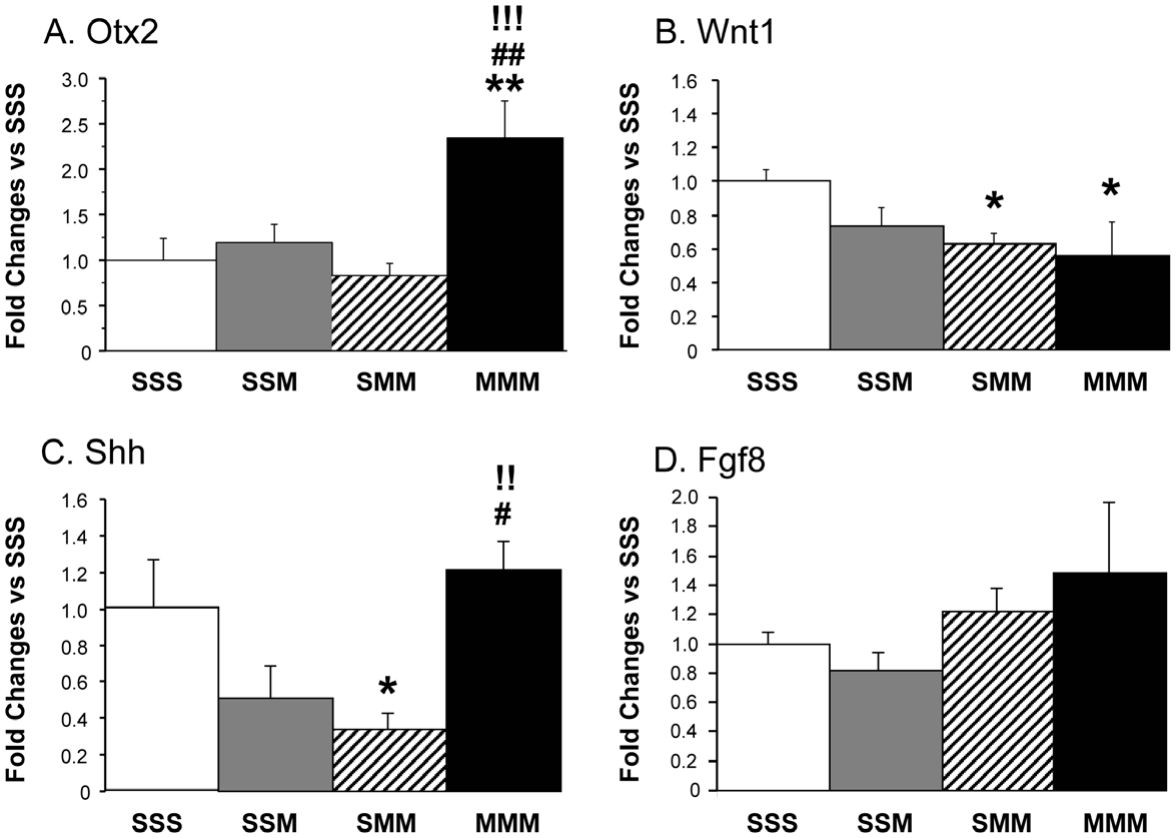
Effect of METH administration on Otx2, Wnt1, Shh and Fgf8 mRNA expression in the midbrain. There were increases in Otx2 mRNA expression only in the METH-preteated group challenged twice with toxic doses of the drug (A). Animals challenged twice with METH showed significant decreases in Wnt1 expression (B). There were significant decreases in Shh expression only in saline-pretreated rats challenged twice with METH (C). METH caused no changes in Fgf8 expression in any of the groups (D). Data were obtained by qRT-PCR using total RNA obtained from 5–6 animals per group. The values represent means ± SEM in comparison to the saline-pretreated challenged with saline group (SSS). N = 5–6 animals per group. Keys to statistics are as described in Fig. 4.

The expression of TH and DAT is regulated during development by a number of transcription and trophic factors including OTX2, WNT1, SHH and FGF8 which are involved in early differentiation of midbrain DA neurons [22], [24]–[26], [47]. Thus, we reasoned that toxic doses of METH might have differential effects on the expression of these factors in the absence or presence of drug preconditioning. Figure 5A shows that METH challenges caused increases in *Otx2* expression in the METH- but not in the saline-pretreated rats. A single METH challenge decreased *Wnt1* mRNA levels but these effects did not reach significance. In contrast, the animals that received two METH challenges (SMM and MMM groups) experienced significant reductions in *Wnt1* expression regardless of pretreatment (Fig. 5B). The single drug challenge also caused non-significant decreases in *Shh* mRNA levels whereas the two challenges led to significant decreases in the saline-pretreated group (Fig. 5C). In contrast, *Shh* mRNA levels were normal in METH-pretreated group after the drug challenges (Fig. 5C). There were no significant changes in *Fgf8* expression in any of the METH-treated groups (Fig. 5D).

#### Effects of METH on the expression of *Lmx* and *Msx* families of transcription factors

**Figure 6:**
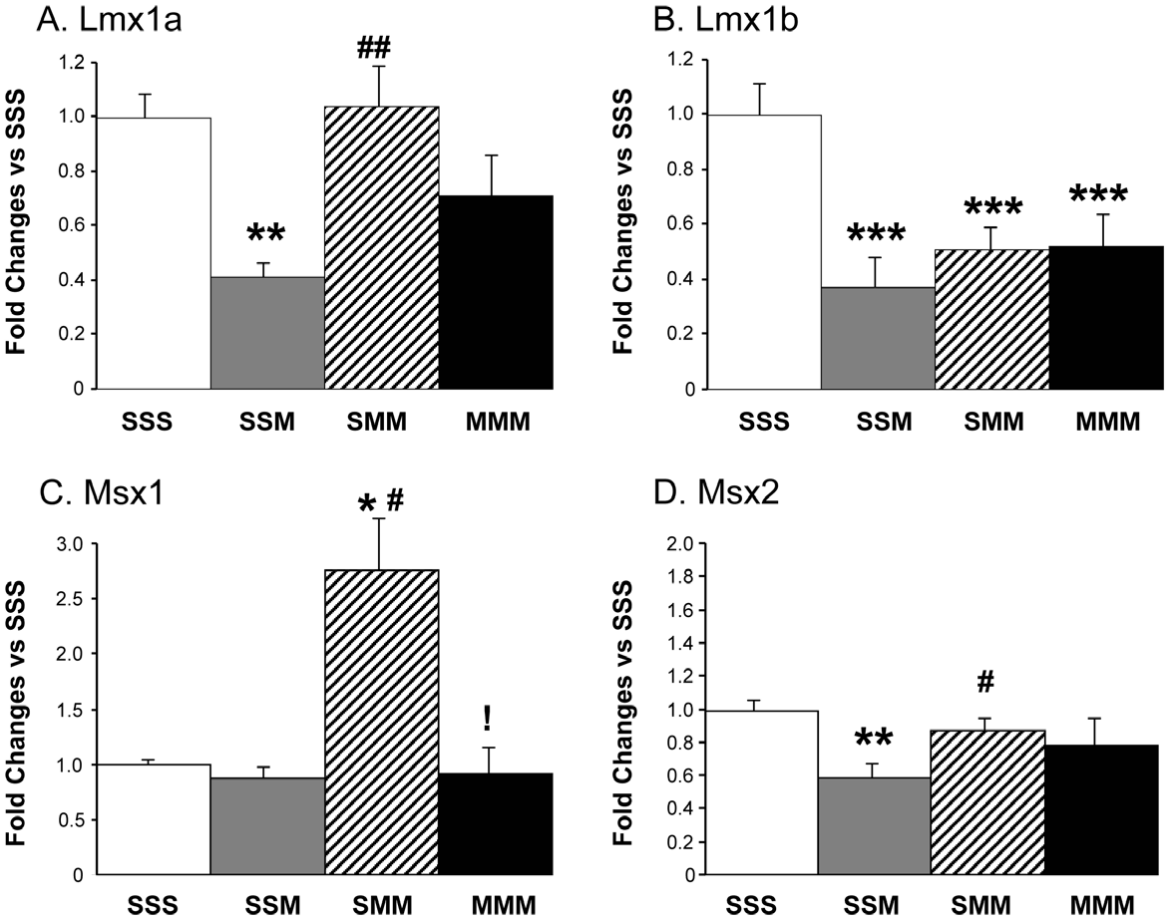
METH treatment induced different changes in Lmx and Msx gene expression in midbrain. METH injections caused significant decreases in Lmx1a expression only in the SSM group (A) but resulted in reductions in Lmx1b mRNA levels in all groups independent of pretreatment (B). There were increases in Msx1 expression only in the saline-pretreated group challenged twice with METH (C). In contrast, METH caused decreases in Msx2 expression in the SSM group (D). The values represent means ± SEM in comparison to the saline-pretreated challenged with saline group (SSS). N = 5–6 animals per group. Keys to statistics are as described in Fig. 4.

Other factors that participate in intermediate steps in the differentiation of DA neurons during development include LMX1A, LMX1B, MSX1, and MSX2 [48], [49]. Figure 6 shows the effects of METH on their expression. *Lmx1a* mRNA levels were decreased only in the group challenged once with METH after saline pretreatment (Fig. 6A). There also comparable decreases in *Lmx1b* expression in all drug-treated groups regardless of pretreatment (Fig. 6B). Interestingly, the two METH challenges caused increases in *Msx1* expression in the saline-pretreated group (Fig. 6C). In contrast, the single METH challenge led to decreases in *Msx2* expression (Fig. 6D) in the saline-pretreated rats whereas other groups were not affected.

#### Effects of METH on midbrain *Nurr1* and *Pitx3* mRNA and protein levels in the midbrain

**Figure 7:**
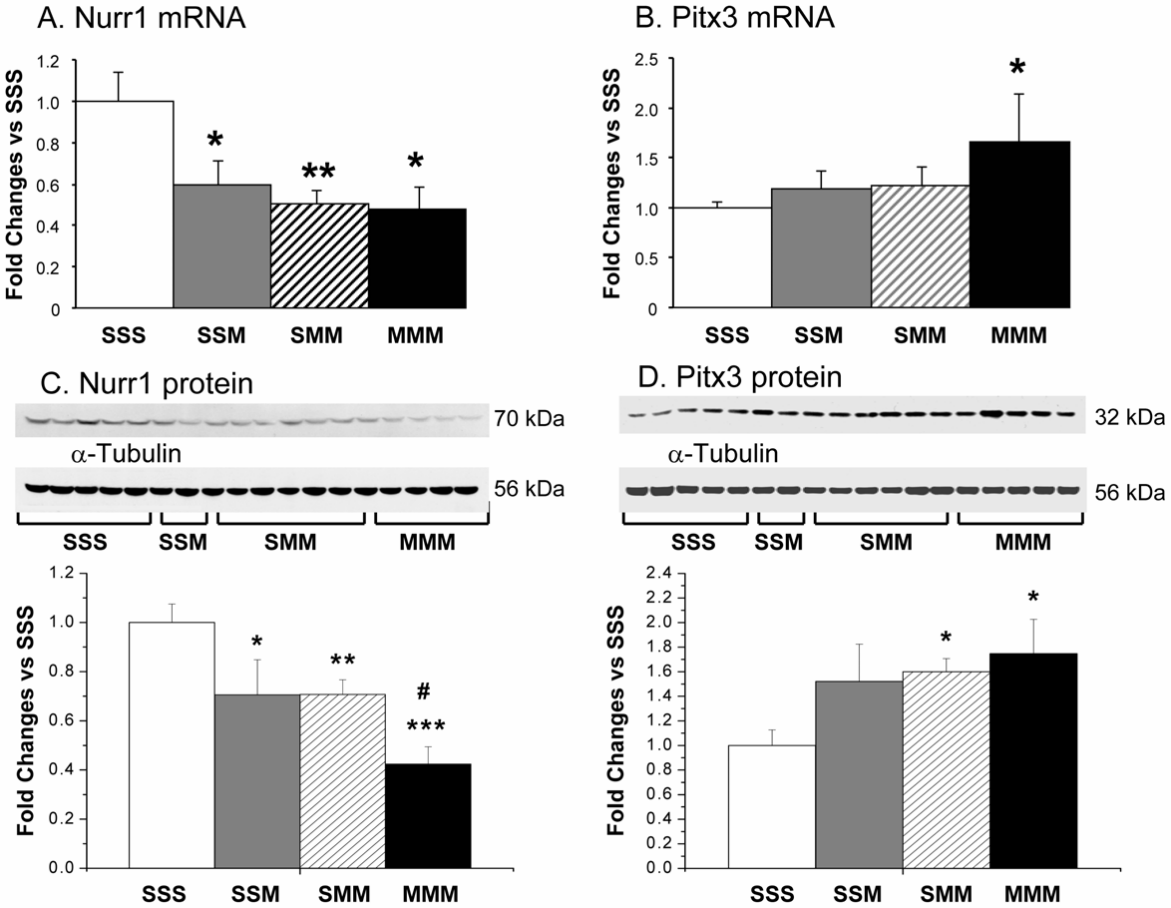
METH caused differential changes in Nurr1 and Pitx3 mRNA and protein levels in the midbrain. METH injections resulted in significant decreases in Nurr1 expression in all the groups (A). In contrast, METH challenges caused significant increases in Pitx3 mRNA expression only in the METH-preconditioned rats (B). Consistent with the mRNA data, toxic METH challenges caused decreases in Nurr1 protein levels in all the groups (C). In contrast, Pitx3 protein levels were increased by METH (D). The values represent means ± SEM in comparison to the saline-pretreated challenged with saline group (SSS). N = 3–6 animals per group. Keys to statistics are as described in the legend to Fig. 4.

We measured the expression of NURR1 and PITX3 because they participate the molecular regulation of TH and DAT levels during both developmental stages and in adult life [50]. Figure 7 presents the effects of METH on their expression in the midbrain. Unexpectedly, METH caused significant and similar decreases in *Nurr1* mRNA levels in the presence and absence of METH pretreatment (Fig. 7A). In contrast, there were METH challenge-induced increases in *Pitx3* expression only in the METH-preconditioned rats (Fig. 7B).

Similar to the effects of METH on *Nurr1* mRNA, there were decreases in midbrain NURR1 protein levels in all the groups (Fig. 7C). Moreover, the decreases observed in the METH-pretreated group were of greater magnitude than those found in the other two groups. METH challenges caused increases in PITX3 protein levels in the midbrain in both groups (SMM and MMM) that received two drug challenges irrespective of pretreatment (Fig. 7D).

## Discussion

Dopaminergic neurons of the ventral midbrain, which includes ventral tegmental area (VTA) and substantia nigra pars compacta (SNpc), play important roles in the control of motor and psychomotor behaviors [1]. Functional and structural pathologies in these systems form the substrates for Parkinson's disease and addictive disorders [51], [52]. In the present study as in other investigations [8], repeated injections of large doses of METH, given within short time intervals, caused decreases in DA, TH, and DAT in the rat striatum. We also showed that a second challenge with METH did not elicit any further decreases in DA markers. The lack of further reductions after the second METH challenge is probably related to the fact that decreases in DAT binding are measurable within 24 hours after injections of toxic doses of METH [8] and to the findings that DAT is an important determinant of METH toxicity [53]. In contrast, chronic intermittent injections of non-toxic METH doses provided almost complete protection against striatal DA depletion and against decreases in TH protein levels induced by toxic METH challenges. Unexpectedly, this pattern of METH pretreatment allowed only partial protection against reductions in striatal DAT protein levels even though *Dat* mRNA expression was robustly increased in the midbrain. These observations suggest that following drug-induced increased synthesis of DAT, the protein might be rapidly degraded by METH-triggered pathways including oxygen-based free radicals [8], [18]. Alternatively, the translation of mRNA into DAT protein or its transport towards striatal DA terminals might be negatively impacted by the METH injections. It is also important to note that, because all animals independent of pretreatment showed significant hyperthermia after the second METH challenge, it is unlikely that the protection caused by METH preconditioning is dependent on changes in temperature regulation.

The substantial loss of DA markers observed in the present study following injections of toxic METH doses is consistent with the observations of other investigators who used similar doses of the drug [9]–[11], [54]–[56], see [8] for review. The differential protection of METH pretreatment against the toxic effects of the drug, preventing the decreases in striatal DA and TH but not the reductions in DAT were unexpected and points to a dissociation of METH effects on these striatal markers. This idea is consistent with recent papers that have documented differential effects of METH administration on DA and DAT in striata of rodents. For example, Xi et al. [57] reported that a single injection of a moderately large dose of METH (20 mg/kg) caused marked decreases in striatal DA levels measured one month after the injection without affecting DAT binding. In contrast, Schwendt et al. [58] found that extended METH self-administration was associated with decreased DAT levels without affecting DA and TH levels in the striatum. The latter findings are almost identical to our observations in the METH-preconditioned rats challenged with toxic doses of the drug. Yet, another study had reported increases in TH mRNA and protein levels in the midbrain at 1 day, but not at 30 days, after cessation of METH self-administration in rats [59]. In contrast, there were no effects of METH on TH protein levels in striatal dopaminergic terminals. Importantly, METH self-administration had no significant effects on *Dat* mRNA levels in the midbrain [59]. Our findings are also consistent with the observations in adult weaver mutant mice that exhibit loss of DA neurons in the nigrostriatal dopaminergic pathway [60]. Specifically, expression of *Dat* mRNA in remaining SNpc dopaminergic neurons was decreased whereas the level of *Th* mRNA was not affected in these animals [60]. Most importantly, however, these observations indicate that the various dopaminergic markers cannot be used interchangeably to study METH toxicity in the rodent striatum.

The differential METH-induced changes in TH, DAT and DA levels could also be secondary, in part, to perturbations in transcription factors that influence the expression of these DA markers. Among these transcription factors, OTX2, WNT1, and SHH are involved in regulating the early development of DA neurons [22], [24]–[26], [61]. Thus, it was of interest that the METH challenges caused substantial increases in *Otx2* mRNA only in the animals pre-exposed to lower doses of METH. These observations suggest that treatment with low non-toxic doses of the drug might have altered the transcription of *Otx2* to such an extent that challenges with large METH doses were able to increase *Otx2* mRNA levels in midbrain DA neurons in METH-preconditioned rats but not in saline-pretreated animals. In contrast to *Otx2* mRNA expression which was not affected by METH in saline-pretreated animals, *Shh* transcript levels were markedly reduced in these rats. SHH is thought to be responsible for ventral fate determination during development [62], being important in the formation, size, and shape of the ventral midbrain [22], [61]. Conditional inactivation of *Shh* after 8 days, but not after 11 days, of gestation causes loss of DA populations [63]. SHH also plays an important role in the formation of DA axonal projections to rostral brain regions including striatum [64]. Thus, the decreased expression of the *Shh* transcript after METH challenges in the two saline-pretreated groups, which show decreased dopaminergic markers, in contrast to normal *Shh* mRNA levels in the METH-preconditioned rats that exhibit normal levels of DA and TH protein, suggest that SHH may play a partial role in maintaining DA homeostasis in the striatum of METH pretreated rats. Although much remains to be done to elucidate the role of SHH by itself or in combination with OTX2 expression in maintaining DA neurons in the adult brain, it is unlikely that either one of them is involved in the METH-induced changes in DAT expression since the drug caused significant decreases in DAT expression in both saline- and METH-pretreated rats, albeit to a lesser extent in the latter than in the former group.

Unexpectedly, we found that METH challenges caused decreases in *Nurr1* mRNA and protein levels in both saline- and METH-pretreated rats. NURR1 is a member of the nuclear receptor superfamily of transcription factors [65] and is very important for the induction and maintenance of DA neurons [28], [29], [66], [67]. The expression of *Nurr1* in the adult brain suggests that it might play additional roles in the nervous system [68], including maintenance of midbrain DA neurons [28]. Therefore, the decreases in *Nurr1* mRNA and protein levels in midbrain after METH challenges both in saline- or METH-pretreated animals are disconcerting because they might render DA neurons more vulnerable to cell death. However, preclinical toxic doses of METH or amphetamine that cause decreases in TH, DAT, and DA levels in the striatum are not associated with DA cell loss in SNpc ([69], [70], reviewed in [8]). This lack of METH-induced death of DA neurons might be related to the fact that toxic METH challenges failed to cause perturbations in *Pitx3* expression in saline-pretreated animals while increasing *Pitx3* expression in METH-preconditioned rats. The importance of PITX3 in the development of midbrain DA neurons is supported by findings that both NURR1 and PITX3 can cooperate to promote the maturation of DA neurons [29] and by observations showing the loss of dopaminergic neurons in the midbrain of *Pitx3*-deficient aphakia mice [71]. Because NURR1 and PITX3 bind to upstream regulatory sequences in *Th* and *Dat* [27], [29], [30], [72], the partial loss of NURR1, without decreases in PITX3 expression, might be enough to cause decreases in striatal TH protein levels but not sufficient to induce retrograde degeneration of midbrain DA neurons. This idea is consistent with the protection against METH-induced decreases in TH expression and DA depletion in the striatum of the METH-preconditioned group which shows increased PITX3 expression in the midbrain.

In summary, we report that toxic doses of METH influence the expression of DA differentiating factors in the absence and presence of drug pretreatment, suggesting that repeated METH injections might trigger substantial adaptive changes in transcriptional responses in the adult mesostriatal dopaminergic system. This is consistent with a notion that the amphetamines can recapitulate developmental processes in the brain [73] since amphetamine conditioned place preference is associated with enrichment of transcription factors that regulate brain development in the zebrafish [74]. Our observations also point to complex regulatory networks involved in the control of DAT and TH expression in the adult brain exposed to METH. These networks need to be considered when developing medications for the treatment of METH addicted individuals. Finally, the possible involvement of these developmental transcription factors in regulating dopaminergic circuitry in the adult brain exposed to other dopaminergic toxins need to be evaluated further.

## Supporting Information

Table S1 Dosing schedule used for METH escalating dose pretreatment and challenge METH injections.
(JPG)
