# Supplementary figures and images for: Chronic Methamphetamine Administration Causes Differential Regulation of Transcription Factors in the Rat Midbrain
Source: PLoS One. 2011 Apr 25;6(4):e19179. doi: 10.1371/journal.pone.0019179 (PMC3081849; doi:10.1371/journal.pone.0019179)

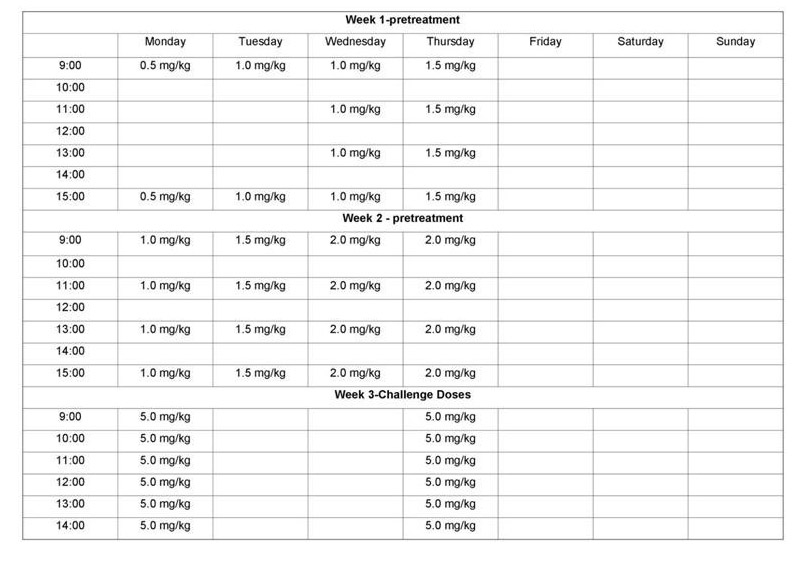

Supplement: Table S1 — Dosing schedule used for METH escalating dose pretreatment and challenge METH injections. (JPG) [file pone.0019179.s001.jpg]
